# Childhood interstitial lung disease due to surfactant protein C deficiency: frequent use and costs of hospital services for a single case in Australia

**DOI:** 10.1186/1750-1172-9-36

**Published:** 2014-03-19

**Authors:** Neil J Hime, Dominic Fitzgerald, Paul Robinson, Hiran Selvadurai, Peter Van Asperen, Adam Jaffé, Yvonne Zurynski

**Affiliations:** 1 Australian Paediatric Surveillance Unit, Kids Research Institute, Westmead, NSW 2145, Australia; 2 Discipline of Paediatrics and Child Health, Sydney Medical School, The University of Sydney Sydney, Australia; 3 Department of Respiratory Medicine, The Children’s Hospital at Westmead, Westmead, NSW 2145, Australia; 4 Department of Respiratory Medicine, Sydney Children’s Hospital, Randwick, NSW 2031, Australia; 5 Discipline of Paediatrics, School of Women’s and Children’s Health, UNSW Medicine, The University of New South Wales, Sydney, Australia

**Keywords:** Rare diseases, Health services, Health costs, Surfactant protein C deficiency, Childhood interstitial lung disease

## Abstract

**Background:**

Rare chronic diseases of childhood are often complex and associated with multiple health issues. Such conditions present significant demands on health services, but the degree of these demands is seldom reported. This study details the utilisation of hospital services and associated costs in a single case of surfactant protein C deficiency, an example of childhood interstitial lung disease.

**Methods:**

Hospital records and case notes for a single patient were reviewed. Costs associated with inpatient services were extracted from a paediatric hospital database. Actual costs were compared to cost estimates based on both disease/procedure-related cost averages for inpatient hospital episodes and a recently implemented Australian hospital funding algorithm (activity-based funding).

**Results:**

To age 8 years and 10 months the child was a hospital inpatient for 443 days over 32 admissions. A total of 298 days were spent in paediatric intensive care. Investigations included 58 chest x-rays, 9 bronchoscopies, 10 lung function tests and 11 sleep studies. Comprehensive disease management failed to prevent respiratory decline and a lung transplant was required. Costs of inpatient care at three tertiary hospitals totalled $966,531 (Australian dollars). Disease- and procedure-related cost averages underestimated costs of paediatric inpatient services for this patient by 68%. An activity-based funding algorithm that is currently being adopted in Australia estimated the cost of hospital health service provision with more accuracy.

**Conclusions:**

Health service usage and inpatient costs for this case of rare chronic childhood respiratory disease were substantial. This case study demonstrates that disease- and procedure-related cost averages are insufficient to estimate costs associated with rare chronic diseases that require complex management. This indicates that the health service use for similar episodes of hospital care is greater for children with rare diseases than other children. The impacts of rare chronic childhood diseases should be considered when planning resources for paediatric health services.

## Background

Chronic disease in children occurs in the context of rapid physical and mental development, necessitating more complex care than in adults [1]. Medical management and treatment planning must take into consideration implications for long-term health and well-being during childhood, adolescence and into adulthood.

Of the 37% of children in Australia living with a chronic disease, 10% are affected by asthma and the rest are affected by a diverse range of conditions [2], many of which are rare (defined by the European Union as affecting less than 5 persons per 10,000 of the population) [3]. Children with rare chronic conditions often require care by multiple paediatric specialists and allied health professionals, necessitating effective coordination of services and service providers.

Rare diseases of childhood present demands on health services and families [4]. A lack of clinical guidelines for many rare conditions, coupled with limited experience and expertise among health professionals in the broader community contribute to the increased burden on services [5]. The burden of rare chronic paediatric disease on families, the health system and the economy is likely to be substantial, however empirical evidence for this is lacking.

Childhood interstitial lung disease (ChILD) is a heterogeneous group of rare childhood respiratory diseases [6,7]. ChILD conditions affect the lung interstitium (the tissue and space around alveoli where gas exchange occurs) and have a different aetiology to that of adults with interstitial lung disease [8]. ChILD associated with an autosomal dominant mutation^a^ in the surfactant protein C (SP-C) gene was first described in 2001 [9]. SP-C has a role in surfactant function to reduce surface tension and prevent atelectasis [10]. SP-C may also have anti-inflammatory properties [11]. Disease severity associated with SP-C deficiency is highly variable, ranging from asymptomatic to severe respiratory distress and deteriorating lung function that is exacerbated by recurring viral and bacterial lung infections [12-17]. The variable natural history of the disease and absence of randomised, placebo-controlled trials or cross-over studies means that optimal therapies for individuals with SP-C deficiency are unknown [15]. Treatment is supportive with nutrition and, oxygen and/or non-invasive ventilation. Drug treatment is focussed on improving lung function through reducing lower airway inflammation with corticosteroids, hydroxychloroquine and azithromycin [16,18,19]. In some cases of severe respiratory failure, lung transplant is the only treatment option [17,20]. Here we describe the health services utilisation and associated financial costs of care of a child with a severe case of chronic ChILD due to SP-C deficiency.

In Australia, costs associated with public hospital-based care are generally covered by the government through Medicare [21]. This includes all non-elective surgery and treatments conducted at tertiary and paediatric hospitals. Hospitals are funded by the government according to the number and type (casemix) of patients that they treat [22]. Central to this system of budgeting hospital costs is classifying each inpatient episode by diagnosis and/or procedure (Australian Refined Diagnosis Related Group (AR-DRG)) [23]. As part of the National Health Reform Agreement 2011 [24] between the Australian Federal Government and individual State Governments, AR-DRG are to be applied to a complex algorithm so that each episode of hospital care is quantified and normalised to a National Weighted Activity Unit (NWAU). The number of NWAUs for each hospital episode is multiplied by a National Efficient Price to determine the estimated cost associated with each episode of care. The National Efficient Price is determined annually by a body that is independent of government, the Independent Hospital Pricing Authority (IHPA) [25]. This funding arrangement is called “activity-based funding”. Activity-based funding is being progressively implemented in Australia from 2012 and is designed to standardise the measurement of resource requirements of hospitals across demographics, geographies and medical specialties.

In this study we have applied activity-based funding calculations to the tertiary, paediatric inpatient episodes of this single patient with ChILD. We compared the actual costs of paediatric inpatient episodes with activity-based funding estimates and with AR-DRG-related cost averages.

## Methods

Health service utilisation data were obtained from a detailed review of the patient’s case notes, hospital records and information provided by managing physicians.

Costs of care were examined for inpatient tertiary hospital episodes only. Actual costs of hospital services were extracted from databases of the Management Support and Analysis Unit, Sydney Children’s Hospitals Network (SCHN)-which comprises of two paediatric hospitals in Sydney, Australia (The Children’s Hospital at Westmead and Sydney Children’s Hospital, Randwick) and Health Information Services, Alfred Health, Melbourne, Australia. Budget estimates of services provided by the SCHN were determined from AR-DRG-associated cost averages for inpatient episodes. AR-DRG’s were assigned by medical coders in hospital Medical Records Departments. Activity-based funding estimates for inpatient health services provided by the SCHN were determined by entering data for each hospital episode for this case into the IHPA 2012–13, NWAU calculator [26].

Consent was obtained from the patient’s mother for the collection and publication of de-identified information relating to the health service use and medical needs of the child. Approval was also obtained from the Research Ethics Committee of the SCHN to conduct this study.

### Analysis

Summed number of presentations to Emergency Departments, hospital admissions, inpatient days in hospital, inpatient days in the paediatric intensive care unit (PICU), outpatient consults and common clinical investigations are described. An admission is defined as a stay in hospital of four hours or more. Hospital services usage is described until the patient was aged 8 years, 10 months. Whereas costs of tertiary hospital inpatient services (SCHN hospitals and a paediatric transplant unit in an adult hospital where the lung transplant was performed) are described until the child was aged 7 years, 9 months.

## Results

### Clinical course and health service use

Following a clomiphene assisted conception, an uneventful pregnancy and delivery of a female infant ensued. The infant was initially admitted to a small regional hospital outside of Sydney at 21 days of age for failure to thrive. Over four days the infant gained weight and was discharged home but returned to hospital in the evening of the day of discharge with respiratory distress. The infant was transferred 164 kilometres by emergency transport to The Children’s Hospital at Westmead in Sydney, a tertiary/quaternary paediatric hospital. The infant remained in this hospital during the next few months, where multiple investigations (bronchoscopy, thoracoscopic lung biopsy, computed axial tomography of the chest and chest x-rays) were performed and a provisional diagnosis of chronic pneumonitis of infancy (a type of ChILD) was determined. A lung biopsy sample was sent to a histopathologist in the USA who specialised in ChILD. Their analysis supported the diagnosis of chronic pneumonitis of infancy and, immunohistochemistry performed by this specialist suggested a surfactant protein deficiency. A mutation in the SP-C gene was confirmed when the infant was 5 months old by genetic test in the USA. Respiratory symptoms were treated with corticosteroids, hydroxychloroquine and continuous oxygen. With continuous nasogastric feeds the infant gained weight and at 3 months of age she was transferred back to the regional hospital for a further month until discharge home on continuous oxygen therapy.

At 7 months of age the infant was admitted to PICU with respiratory failure and severe nocturnal hypoxic episodes, discovered during a scheduled sleep study. Bi-level positive airway pressure (BiPAP) non-invasive ventilation was commenced. Gastro-oesophageal reflux and poor feeding required laparoscopic fundoplication and gastrostomy. A chest infection required intubation. At 14 months of age the infant was discharged on continuous oxygen therapy, nocturnal BiPAP and support from a community health care team (case manager, nurse, general paediatrician) was arranged.

Respiratory function slowly and progressively declined despite pharmacotherapy, oxygen and BiPAP. Pancreatic insufficiency causing chronic fatty diarrhoea (not known to be associated with ChILD) responded to regular pancreatic enzyme replacement therapy. A sweat test excluded cystic fibrosis as the cause of the pancreatic insufficiency. The respiratory deterioration accelerated following a respiratory syncytial virus infection at 4 years of age that required an emergency transfer from the regional hospital to Sydney Children’s Hospital and a 14 day stay in PICU. At 6 years of age the child had end stage respiratory failure and pulmonary artery hypertension, and was wheelchair bound. She continued to have an aversion to oral feeds and was gastrostomy fed. Proximal muscle weakness and mild developmental delay required physiotherapy and speech therapy. She had mid-face hypoplasia, partly attributed to the use of non-invasive face mask ventilation from late infancy.

At age 6 years and 10 months the child underwent a bilateral, sequential lung transplant in a paediatric transplant unit in an adult tertiary/quaternary hospital (The Alfred Hospital, Melbourne, Australia). Four months post-transplant the child no longer required respiratory support and nine months post-transplant she could exercise without limitation. Post-operative medical issues that were resolved included: tachycardia, atrial arrhythmia, thrombosis, vocal cord palsy and *stenotrophomonas maltophilia* colonisation of the airways.

Ongoing medical issues at 8 years and 10 months of age were: aversion to oral feeds, leakage of feeds from the gastrostomy site, complications from chronic use of corticosteroids (glucose intolerance, osteoporosis and hypertrichosis), mid-face hypoplasia and malocclusion (related to the long term use of non-invasive face mask ventilation), speech and language delay, lifelong immunosuppression for Epstein-Barr Virus mismatched donor lungs, medication-related leukopenia, bronchial anastomosis stenosis requiring repeated dilatation by bronchoscopy, pseudomonas colonisation of the lungs and intermittent morning nausea.

### Use of hospital services

The child had 32 hospital admissions and spent more than one year (443 days) of her first 8 years and 10 months of life in hospital for management of ChILD and associated respiratory infections, including 298 days in PICU (Table 1). This is an average of 50 days per year of life spent as a hospital inpatient.

**Table 1:** Hospital admissions, presentations to emergency departments and outpatient clinic attendances

**Admissions**	
Tertiary hospital admissions	
Paediatric hospital managing the condition of ChILD	22
Other paediatric hospital	1
Adult hospital with paediatric lung transplant unit	4
Regional hospital admissions	5
**Total**	**32**
**Reasons for hospital admissions**	
Respiratory distress	7
Blood gas monitoring	4
Multi-disciplinary review	6
Intravenous immunoglobulin	4
Lung biopsy	2
Respiratory infection	2
Respiratory airway dilation (post-lung transplant)	5
Lung transplant	1
Sleep study (scheduled)	1
**Inpatient days**	
Days as an inpatient of tertiary hospitals	
Paediatric hospital managing the condition of ChILD	337
Other paediatric hospital	14
Adult hospital with paediatric lung transplant unit	33
Days as an inpatient of the regional hospital	61
Total*	**443**
**Total number of days in PICU**^***a***^	**298**
**Presentations to Emergency Departments**	**10**
**Direct transfers between regional and tertiary hospitals**	**3**
Outpatient attendances**	
Tertiary hospital clinics	18
Regional hospital clinics	9
**Total**	**27**

*The total is less than the sum of tertiary and regional hospital inpatient days because on two occasions the patient was an inpatient of two hospitals on the same day.

**Does not include outpatient clinic consults while the patient was an inpatient.

^*a*^PICU, Paediatric Intensive Care Unit.

**Figure 1:**
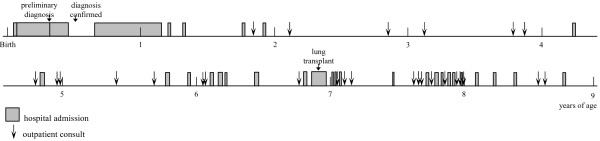
**Timeline of hospital service use for a single patient with ChILD**^***a***^***.*** Shown on the timeline are all hospital admissions and attendances at hospital outpatient clinics including: tertiary paediatric hospitals, a tertiary adult hospital (for a lung transplant) and a small, regional hospital. The length of stay for each hospital admission is represented by the width of the shaded block. Attendances at hospital outpatient clinics do not include consults at outpatient clinics while the patient was an inpatient. The unit of the time line is the patient’s age in years. ^*a*^ChILD, childhood interstitial lung disease.

Three lengthy hospital stays accounted for the majority of inpatient service use and occurred mainly during the first 2 years of life (Figure 1). Firstly, a period of 126 days in the first few months of life to stabilise respiratory function and, to determine a preliminary diagnosis and management plan. Two direct transfers between the regional hospital and paediatric hospital in Sydney (The Children’s Hospital at Westmead) occurred during this time. Secondly, there was a 199 day admission to manage a critical deterioration of respiratory function before the child’s first birthday. Thirdly, in her seventh year the child spent 30 days in hospital for a lung transplant. Among the total of 32 hospital admissions, seven were for respiratory distress, two related to acute respiratory infections and all other admissions were for investigations or procedures associated with the management of ChILD (Table 1). In addition, there were 27 consults at hospital outpatient clinics.

Intensive, ongoing treatment (pharmacotherapy, oxygen and BiPAP) coincided with a reduction in the use of hospital services between the ages of 2 and 4 years. From 4 years of age respiratory function declined, resulting in an increase in the use of inpatient services. The lung transplant at age 6 years and 10 months along with post-operative monitoring and treatment accounted for a further increase in hospital services use. At age 8 years the child’s condition was considerably improved and whilst hospital service use had decreased she continues to be seen every two to three months for ongoing post-lung transplant care.

At least 30 different health professionals were involved in the hospital-based care and disease management (Table 2). Experts in the USA, UK and Germany were also consulted.

**Table 2:** Hospital-based disciplines involved in the patient’s care and management of ChILD

General paediatrician	Radiologist
Paediatric respiratory physician (including USA*)	Neurophysiologist
Paediatric sleep physician	Neurologist
Neonatologist	Transplant specialist (including USA*)
Emergency physician	Immunologist
Nurse practitioner	Transplant nurse
Respiratory technician	Dermatologist
Clinical nurse consultant	Pharmacist
Occupational therapist	Speech therapist
Physiotherapist	Cardiologist
Ophthalmologist	Gastroenterologist
Dietician	Endocrinologist
Haematologist	Microbiologist
Anaesthetist	Geneticist (including USA*)
Surgeon	Histopathologist (including USA*)

*Expertise was utilised from physicians, geneticists and histopathologists in the USA, UK and Germany.

Numerous investigations were conducted at The Children’s Hospital at Westmead including a total of 58 chest x-rays for diagnosis, disease monitoring and to guide clinical procedures. Two lung biopsies were performed for diagnostic purposes. Bronchoscopies (total = 9) were performed to monitor for signs of donor lung rejection (transbronchial biopsy) or lung infection (bronchoalveolar lavage) or, to dilate the bronchial anastomotic stenosis that had developed post-lung transplantation. Lung function tests (total = 10) and sleep studies (total = 11) were regularly performed pre-lung transplant to monitor disease progression. Many investigations were not scheduled prior to admission but performed as a component of disease management during a hospital stay. For example only one of the 11 sleep studies was scheduled prior to admission. In addition to the lung transplant, surgical procedures included gastrostomy and laparoscopic fundoplication. Seventeen different medications prescribed for the treatment of ChILD, complications of ChILD and other medical issues arising from treatment were needed either continuously or intermittently over several years. The medications included: hydroxychloroquine, corticosteroids and anti-cholinergics to treat respiratory symptoms; antibiotics and antivirals to treat respiratory infections; immunosuppressants to prevent rejection of the transplanted lungs. Medications were also required to treat pancreatic insufficiency, pulmonary hypertension, arrhythmia, nausea and mineral deficiency.

Prior to the lung transplant the child required continuous home oxygen therapy and nocturnal BiPAP ventilation for more than 2,000 days. Oxygen cylinders were regularly supplied via the regional hospital. A State Government service (EnableNSW) provided the family with two BiPAP ventilators and paid for the home oxygen. The Children’s Hospital at Westmead provided manufacturing and fabrication support for medical equipment from time to time.

### Inpatient tertiary hospital service costs

The total cost of inpatient services provided by tertiary hospitals (paediatric and adult) until the patient was aged 7 years and 9 months was $966,531 (Australian dollars), including $900,201 for paediatric hospitals (Table 3). An estimate of paediatric inpatient service costs based on AR-DRG-associated cost averages (budget estimate) was only $284,297, underestimating the actual costs by 68%. Activity-based funding estimates for inpatient services at the paediatric hospitals, based on the National Efficient Price 2012–13, totalled $1,201,996. This overestimate of $301,795 on the actual costs is likely due to the application of the current National Efficient Price (2012–13) to hospital encounters for the previous eight years, when service costs were lower.

**Table 3:** Costs of tertiary hospital inpatient services until the patient was aged 7 years and 9 months (11 months post-lung transplant)

**Service provider**	**Actual costs**	**Budget estimate**	**Activity-based funding estimate**
	*Australian dollars*		
Paediatric hospitals	900,201	284,297	1,201,996
Adult hospital	66,330		
**Total**	**966,531**		

## Discussion

This case study demonstrates the frequent use of tertiary paediatric inpatient services, secondary general inpatient services and, outpatient services associated with the management of a rare chronic lung disease. The tertiary paediatric inpatient service costs of disease management were extremely high at AU$ 900,201 over eight years for this one case.

The child in this case study spent 443 days as a hospital inpatient during 32 admissions in her first 8 years and 10 months of life, an average of 50 days as an inpatient per year. In Australia in 2011–12 the total number of inpatient days for children aged 0–9 years was approximately 1,475,000 [27], amounting to an average of 0.5 patient-days per child per year based on a population of 2,908,425 children (0–9 years) [28]. Thus the inpatient health service requirements for the child in this study are one hundred times greater than the needs of an average Australian child.

Over 30 different health professionals were involved in the child’s care. A significant proportion of the health service cost occurred in the first two years, when a management strategy was being devised for a rare disease for which clinical guidelines were not available. The patient developed an aversion to oral feeding as a result of poor lung function and this required a gastrostomy and its ongoing management; chronic treatment with hydroxychloroquine required ophthalmologic monitoring for macular toxicity; chronic use of non-invasive ventilation resulted in mid-face hypoplasia, impairing jaw and tooth development and causing further problems with breathing and speech. Management of these adjunct health issues greatly contributed to the use and cost of health services.

The chronicity and complexity of rare childhood conditions requires management by many highly trained specialists, a variety of specialist services and, other health professionals thereby impacting on health resources [4,29]. A number of reports have shown that children with chronic and complex conditions contribute significantly to paediatric health service burdens and costs [4,30,31]. Colvin and Bower reported that the length of hospital stay is greater in children with complex birth defects than other children, for hospital admissions for the same diagnosis [30]. A retrospective cohort analysis in 37 United States tertiary paediatric hospitals found that patients with more than three readmissions in one year accounted for only 2.9% of all patients admitted, but 18.8% of all admissions and 23.2% of all hospital charges [31]. In a population-based study in the United States of children with chronic conditions, the 5% that were categorised as *severe* accounted for 33% of all hospital admission days resulting from chronic disease [32].

Estimated costs of paediatric inpatient services for this patient, based on the AR-DRG-related cost averages used in Australia prior to 2012, were 68% less than actual costs. This demonstrates the limitation of DRG classifications to estimate health service costs associated with rare chronic and complex paediatric conditions. The DRG classification system relies on accurate coding assigned to each inpatient episode. This may be difficult to achieve for rare diseases that do not easily fit into an identifiable classification and where there are multiple morbidities. Acute episodes of illness in children with underlying chronic disease tend to be more complex and severe and therefore more costly when compared with the same acute care in children without underlying chronic complex conditions [33]. In a previous analysis of paediatric inpatient costs, the actual costs of specialist expert care in paediatric tertiary/quaternary hospitals has been underestimated by 77% when using the AR-DRG casemix system due to the large number of comorbidities and complications affecting admitted children [34]. This is similar to the 68% underestimate based on AR-DRG-related cost averages estimated for our single case.

In Australia, the recently implemented activity-based funding model of resource allocation uses an algorithm that incorporates not only AR-DRG diagnostic/procedural codes but also other variables such as hospital level of specialty, length of stay, hours in intensive care and a paediatric weighting [25]. Applying the activity-based funding model in our case study overestimated costs by 33%. This was expected because historical data were entered into the 2012–13 version of the model, which is based on current costs. The activity-based funding model appears to provide a closer approximation to actual inpatient costs for our case than the AR-DRG-related cost averages estimate.

Our study is limited to a description of a single, very severe case requiring lung transplant that does not represent all children with rare chronic lung diseases. Other rare, complex and chronic childhood diseases are likely to have significant impacts through frequent use of multiple services and associated high costs [4]. We only analysed the direct costs of hospital admissions. Costs were not analysed for: medications (chronic use of 17 medications), allied health services, pathology and imaging outside of the hospital system, admissions to a regional hospital (61 inpatient days), outpatient consultations and emergency transport (164 km between regional and paediatric hospitals). These additional costs are likely to be substantial and would have impacted on the Australian health care system. Furthermore, out-of-pocket health and non-health costs to the family have not been accounted for.

Cost savings may be possible when dealing with complex and chronic conditions through treatment discovery, development and implementation of evidence-based clinical guidelines, coordination and integration of multiple health services, and education of health professionals [35-37]. High quality research evidence and clinical trials are needed to support such developments.

## Conclusion

The case described here, and other literature, suggests that although rare chronic conditions may be few in number, they have enormous impacts on health costs and that these costs are likely to be underestimated. This needs to be taken into account when planning and funding health services. The new Australian system of resource allocation (activity-based funding) is likely to more accurately estimate costs associated with rare complex chronic childhood diseases than disease or procedure associated cost averages. Further evidence on large groups of diverse rare diseases is needed to support appropriate resourcing of health services to cover the real costs of rare chronic diseases to the health system. We are currently undertaking a large study funded by the Australian Research Council (Grant LP 110200277) to address this gap in knowledge.

## Endnote

^a^Mutations in the SP-C gene can also arise *de novo* and this was the case for the mutation in this case study as neither parent had the mutation.

## Abbreviations

AR-DRG: Australian Refined Diagnosis Related Group; BiPAP: Bi-level positive airway pressure; ChILD: Childhood interstitial lung disease; IHPA: Independent Hospital Pricing Authority; NWAU: National weighted activity unit; PICU: Paediatric intensive care unit; SCHN: Sydney Children’s Hospitals Network; SP-C: Surfactant protein C.

## Competing interests

The authors declare that they have no competing interests.

## Authors’ contributions

NJH contributed to the design of the study, data acquisition, analysis and interpretation of the data and, drafted the manuscript. DF critically reviewed the manuscript for clinical content and contributed to data acquisition. PR critically reviewed the manuscript for clinical content. HS critically reviewed the manuscript for clinical content. PVA critically reviewed the manuscript for clinical content. AJ critically reviewed the manuscript for clinical content. YZ conceived the study, contributed to the analysis and interpretation of the data and, reviewed the manuscript for intellectual content. All authors read and approved the final manuscript.

## Authors’ information

NJH, Australian Paediatric Surveillance Unit, Kids Research Institute, Sydney, Australia; Discipline of Paediatrics and Child Health, Sydney Medical School, The University of Sydney, Sydney, Australia. DF, Department of Respiratory Medicine, The Children’s Hospital at Westmead, Sydney, Australia; Discipline of Paediatrics and Child Health, Sydney Medical School, The University of Sydney Clinical School, Sydney, Australia. PR, Department of Respiratory Medicine, The Children’s Hospital at Westmead, Sydney, Australia; Discipline of Paediatrics and Child Health, Sydney Medical School, The University of Sydney Clinical School, Sydney, Australia. HS, Department of Respiratory Medicine, The Children’s Hospital at Westmead, Sydney, Australia; Discipline of Paediatrics and Child Health, Sydney Medical School, The University of Sydney Clinical School, Sydney, Australia. PVA, Department of Respiratory Medicine, The Children’s Hospital at Westmead, Sydney, Australia; Discipline of Paediatrics and Child Health, Sydney Medical School, The University of Sydney Clinical School, Sydney, Australia. AJ, Department of Respiratory Medicine, Sydney Children’s Hospital, Sydney, Australia; Discipline of Paediatrics, School of Women’s and Children’s Health, UNSW Medicine, The University of New South Wales, Sydney, Australia. YZ, Australian Paediatric Surveillance Unit, Kids Research Institute, Sydney, Australia; Discipline of Paediatrics and Child Health, Sydney Medical School, The University of Sydney, Sydney, Australia.
